# Coordination of a fresh agricultural product supply chain with option contract under cost and loss disruptions

**DOI:** 10.1371/journal.pone.0252960

**Published:** 2021-06-09

**Authors:** Nana Wan, Li Li, Xiaozhi Wu, Jianchang Fan

**Affiliations:** 1 School of Economics and Management, Southwest University of Science and Technology, Mianyang, PR China; 2 Zhongshan Institute, University of Electronic Science and Technology of China, Zhongshan, PR China; 3 School of Marketing and Logistics Management, Nanjing University of Finance & Economics, Nanjing, PR China; Wageningen University, NETHERLANDS

## Abstract

This paper analyzes the option coordination problem of a fresh agricultural product supply chain under two supply chain structures, when the production cost and the loss rate are disrupted simultaneously. This paper provides the explicit option coordination conditions for the disrupted supply chain under two supply chain structures, and then explores the effects of the disruptions and supply chain structure on the option coordination conditions. The results suggest that it is unfavorable to apply the original coordinating contracts without disruptions to coordinate the disrupted supply chain. The coordination of the disrupted supply chain can be achieved with knowledge of the distribution of demand. In two coordinating contracts for the disrupted supply chain, the exercise price is still at the original level without disruptions while the option price deviates from the original level without disruptions. Moreover, the relationships of the coordination conditions in two supply chain structures depend on the value of the profit allocation coefficient. When the profit allocation coefficient exceeds (falls behind) a certain threshold, the option price is set at a higher (lower) value in the supplier-led supply chain structure than in the distributor-led supply chain structure, while the exercise price is set at a lower (higher) value in the supplier-led supply chain structure than in the distributor-led supply chain structure. Finally, the disrupted supply chain with any supply chain structure will perform better in the modified coordinating contracts than in the original coordinating contracts without disruptions.

## 1. Introduction

Fresh agricultural products, such as live seafood, fresh meat, fresh vegetables and fresh fruits, are basic necessities for people and play a remarkable role in the market. The high-efficient operation of supply chains is of great significant in ensuring market supply and price stability for fresh agricultural products. However, managing a fresh agricultural product supply chain is full of challenges due to its nature and high risks. By comparing with canned, frozen and dried agricultural products, fresh agricultural products not only have innate feature of shorter life cycle, but also have new feature of serious circulation loss. For example, the loss rate of fresh agricultural products is up to 15% in many developed countries [1]. In China, 25%-30% of vegetables and fruits are deteriorated in the process of transportation, at wholesale and retail markets [2]. Moreover, fresh agricultural product supply chains are more susceptible to the disruptions of the production cost and the loss rate caused by natural and man-made factors. For example, the epidemic of African swine fever in 2019 severely threatened the output of pork in China, and caused a very large rise in the production costs of pork. Heavy snowfall in 2008 caused vandalism and traffic chaos in large tracts of China, and induced an increased loss rate of fresh vegetables and fruits. Handling disruptions in an efficient way is becoming increasingly important to the operation of fresh agricultural product supply chains.

Option contract, as an effective tool for hedging risks, has already been introduced into fresh agricultural product supply chains. For example, option contract has been applied in the Australian supermarket fruit supply chains [3]. In the United States, weather derivatives (options, futures and combinations) have become a useful hedging tool for rain- and heat-based weather risk in agricultural product supply chains [4]. Option contract offers the buyer the right (not the obligation) for purchasing one more unit of fresh agricultural products by a specific day at a pre-negotiated exercise price subject to paying an option premium to the supplier ahead of time. This contract brings the flexibly to the buyer without doing harm to the supplier. In the academic research, option contract has been shown to mitigate the impact of demand uncertainty and reduce the loss in the process of circulation [5]. However, the derived strategies for managing the fresh agricultural product supply chains are stationary, which may become invalid in a disrupted setting. In this context, this paper studies how to design the option coordination mechanism for a disrupted fresh agricultural product supply chain.

This paper considers the interaction between one supplier and one distributor within the framework of a fresh agricultural product supply chain. It is all known that a fresh agricultural product supply chain has the supplier-led supply chain structure and the distributor-led supply chain structure based on the distribution of power among the members. In the supplier-led supply chain structure, the supplier has a larger market share and acts as the leader firm of the supply chain. In the distributor-led supply chain structure, the distributor is much closer to the market and plays as the core role of the supply chain. For example, a major supermarket has a substantial role in arranging the contract and affecting the demand in Australian retail fruit market [3]. In the UK agri-food industry, 50% of products are sold through a small group of distribution centers [6]. The powerful member has more right to design the contract and even may impose restrictions on the vulnerable member. For example, the rock-bottom prices are paid by the grocery retailers to the upstream suppliers in exchange for the guaranteed supply and quality levels in the UK agri-food industry. Most of the previous literature has only involved a stationary fresh agricultural product supply chain with the supplier-led supply chain structure. This paper studies a disrupted fresh agricultural product supply chain with the supplier-led and distributor-led supply chain structures. The aim of this paper is to develop the option coordination mechanism for a fresh agricultural product supply chain with two supply chain structures when the production cost and the loss rate are disrupted simultaneously. More precisely, this paper deals with the key questions as follows.

1. What impact do the cost and loss disruptions have on the integrated decision of the fresh agricultural product supply chain?
2. How to coordinate the disrupted fresh agricultural product supply chain through option contract under two supply chain structures?
3. What is the effect of the cost and loss disruptions on the option coordination conditions?
4. How does the supply chain structure affect the option coordination conditions?

This paper makes two contributes as follows. (1) To the best of authors’ knowledge, most of previous literature only involved the application of option contract in the stationary fresh agricultural product supply chain without disruptions, where the supplier plays the leader role and has the right to design the contract. This paper extends the existing research by looking at the disruptions of the production cost and the loss rate. Meanwhile, this paper incorporates two supply chain structures in the analysis framework. (2) In addition to the impact of the cost and loss disruptions, this paper explores the impact of supply chain structure on the option coordination conditions. It complements to the existing literatures that do not investigate the impact of supply chain structure on the disrupted fresh agricultural product supply chain.

The remainder of this paper is organized as follows. Related literatures are reviewed in Section 2. Sections 3 and 4 analyze the option coordination policies for the fresh agricultural product supply chain under two supply chain structures without and with disruptions. Section 5 explores the impact of supply chain structure on the option coordination conditions for the disrupted fresh agricultural product supply chain. Section 6 provides a numerical example to elaborate our findings. Section 7 concludes this paper and points out the possible extensions. All the proofs are provided in the Appendix.

## 2. Literature review

Fresh agricultural product supply chain management is an important research field of supply chain management. Cai *et al*. [7] designed a new scheme that includes a wholesale-market clearance contract between the producer and the distributor and a wholesale-price-discount sharing contract between the producer and the third party logistic provider to coordinate a three-level fresh product supply chain. Qin *et al*. [8] proposed the model of the pricing and lot-sizing for fresh produce and food, where the quality and physical quantity deteriorate in the form of time proportion and the demand rate is related with the quality, the selling price and the stock level on display. Hou and Liu -[9] analyzed the coordination problem of a typical Chinese fresh product supply chain, the feature of which is that the supply chain is connected by a wholesale market and the market price is random due to uncontrollable supply and demand. Yan *et al*. [10] introduced the Internet of Things into a three-stage fresh agricultural product supply chain consisted of a manufacturer, a distributor and a retailer to investigate the supply chain coordination with an improved revenue-sharing contract. Wang *et al*. [11] examined the impacts of both carbon trading and refrigerated logistics services on a fresh food supply chain, and further designed a transfer payment mechanism to coordinate the interests of the supplier and the retailer. Yang *et al*. [12] studied the optimal freshness-keeping and pricing decisions of a fresh product supply chain with a supplier and a retailer under the retail mode, the dual-channel mode and the O2O mode. They found that the dual-channel mode brings the highest supplier’s profit while the O2O mode brings the highest consumer surplus. Ye *et al*. [13] investigated the effects of yield and demand risks as well as the farmers’ risk aversion on the production and pricing decisions of an agricultural supply chain formed by contract farming scheme. Yan *et al*. [14] proposed two coordinating contracts based on revenue sharing and wholesale price for a fresh agricultural product supply chain with considering the strategic behavior of consumers.

The impact of supply chain structure has been often discussed within the framework of a fresh agricultural product supply chain. Xiao and Chen [15] investigated the optimal order quantity, shipping quantity and retail price for a fresh product supply chain in the pull (distributor-led) and push (producer-led) models. They showed that both the members and the chain will perform better in the pull model than in the push model. Qian *et al*. [16] applied the three-stage revenue sharing contract model to explore the profit allocation and channel coordination problems for a dairy supply chain. Wu *et al*. [17] studied the product order quantity and selling price for a fresh product distributor and the logistics service level and price for a 3PL service provider under three supply chain structures. They explored the impact of supply chain structure on contract design, each firm’s decisions and channel performance, and then developed two new incentive schemes to coordination the fresh product outsourcing logistics channel. Yu *et al*. [18] proposed two game models to compare the pricing and service level decisions and the profits of a three-stage fresh agri-product supply chain with a supplier, a retailer, and a 3PL provider under two supply chain structures. Zhang *et al*. [19] explored the optimal freshness-keeping effort, order quantity and retail price of a distributor and the optimal shipping quantity of a producer within the framework of a fresh product supply chain in the pull (distributor-dominated) and push (producer-dominated) settings. They found that the distributor will exert a greater effort into preserving the product quality in the pull model than in the push model, while the profits of the members and the channel are larger in the pull model than in the push model. However, all these papers mainly focused on a stationary setting and did not involve option contract.

Disruption management is an attractive research field of supply chain management over the past few years. Cao *et al*. [20] developed the revenue sharing coordination mechanism for a supply chain with one manufacturer and multiple competing retailers when the market demand and the production cost are disrupted simultaneously. Zhang *et al*. [21] designed a contract with a wholesale price, a direct channel’s price and a lump sum fee to coordinate a dual-channel supply chain when either the market demand or the production cost is disrupted. Liu *et al*. [22] analyzed the effect of the demand disruption and three coordination modes on a logistics service supply chain with a logistics service integrator and two logistics service providers. Han *et al*. [23] explored the collection channel and production decisions of a closed-loop supply chain with remanufacturing cost disruption from the perspectives of firm profit and system robustness. Huang and Wang [2] considered that the manufacturer is willing to license the third party to conduct remanufacturing activity. They studied the pricing and production decisions of a closed-loop supply chain when the demand and the supply quantity of used products are disrupted. Rahmani and Yavari [24] investigated the pricing, greening and production decisions for a dual-channel green supply chain under demand disruptions. Zhao *et al*. [25] analyzed the pricing, quality, production decisions and the coordination of a fashion supply chain under demand disruptions, and discuss the design of the government’s incentive policy to promote a fashion firm’s quality improvement.

Disruption management has been introduced into fresh agricultural product supply chain. Sun [26] proposed a two-part tariff contract to coordinate a fresh agricultural product supply chain under supply disruptions. MacKenzie and Apte [27] developed a model of a disruption to quantify the elements of making fresh produce supply chains vulnerable to the disruptions and benefits of different disruption management strategies. Behzadi *et al*. [28] investigated the effectiveness of a mixed set of robust and resilient strategies for a perishable multi-period multi-commodity agribusiness supply chain to manage rare high-impact harvest time and yield disruptions. Huang *et al*. [29] developed a Stackelberg game model for a food supply chain with production disruption to study the impacts of forward and backward integration strategies on the optimal decisions and profits of the supply chain members. Yan *et al*. [30] proposed an improved revenue-sharing contract to coordinate the RFID-based fresh agricultural product supply chain after demand disruption, and explored the impact of RFID application and supply chain coordination on the corporate profits, the social responsibility, and the environmental responsibility. However, all these papers mainly focused on a specific supply chain structure and did not involve option contract.

The application of option contract for hedging supply chain risks has attracted more and more interest of scholars. Zhao *et al*. [31] applied a cooperative game approach to analyze the option coordination problem of a supply chain consisted of a manufacturer and a retailer. They suggested that option contract can coordinate the supply chain with Pareto-improvement, compared to wholesale price contract. Fu *et al*. [32] analyzed the joint pricing and portfolio purchasing policies for one firm with random and price dependent demand in a multi-period setting, where the firm can purchase the products from multiple option contracts and the spot market. They found that the optimal portfolio purchasing policy is of an order-up-to type with a sequence of decreasing thresholds and the optimal reservation quantity and selling price are decreasing in the starting inventory level under the additive demand function. Hu *et al*. [33] explored the optimal purchasing policy for the retailer and the optimal production policy for the manufacturer with option contract and partial backordered, in which the retailer’s demand, the manufacturer’s production yield and instant price are all stochastic. Liu *et al*. [34] used a modified newsvendor model to investigate the optimal capacity investment, option price and exercise price for the express delivery provider and the option coordination condition for the delivery service supply chain. Tao *et al*. [35] considered the capacity pricing problem for the air cargo carrier and the capacity reservation problem for the freight forwarders in the air cargo freight industry, where the carrier offers option contract to multiple freight forwarders. Sharma *et al*. [36] proposed a behavioral model of fairness to explore the fairness concerns of the members in a two-stage supply chain, where option contract is applied by the retailer to purchase products from the supplier. Liu *et al*. [37] applied the conditional value-at-risk (CVaR) criteria to study the option pricing, ordering and production policies and the channel coordination conditions of a supply chain with a risk-neutral supplier and a risk-averse retailer under two supply chain structures. Wan *et al*. [38] analyze the inventory purchasing model with option contracts and a spot market for a manufacturer with an objective of minimizing risk and a constraint on profit target.

Option contract has already been introduced into fresh agricultural product supply chains. Wang and Chen [5] explored the option ordering policy of the retailer, the option pricing policy of the supplier and the option coordination policy of the fresh produce supply chain under the wholesale price and option portfolio contracts. They indicated that the supplier’s optimal joint option and exercise pricing policy does not exist, while his optimal option or exercise pricing policy exists under a given exercise or option price. As the option or exercise price increases toward the optimum, the supplier will obtain more profit while the retailer will obtain less profit. Yang *et al*. [39] introduced call, put and bidirectional option contracts provided by the supplier into an agricultural supply chain with sale effort dependent demand. They found that the optimal firm and options order quantity will increase with the sales effort and the option price will balance the impact of loss rate on the channel coordination. Among these three option contracts, the purchase price of bidirectional option is the highest, the order quantity of bidirectional option is the least, and the order quantity of put option is the highest. Zhou *et al*. [40] investigated the option coordination policy for a fresh agri-food supply chain under uncertain demand. They indicated that option contract can prompt the retailer to share demand information with the producer and can help maintain the strategic cooperation between two members. Yan *et al*. [41] analyzed the ordering and coordination problem of a fresh agricultural product supply chain based on two-period price, wholesale price and option contract. However, all these papers mainly focused on a specific supply chain structure and did not involve a disrupted setting.

From above, it is easy to find that few studies have incorporated multiple research fields on disruption management, supply chain structure and option contract within the framework of a fresh agricultural product supply chain. This paper fulfills the gaps of the existing related literature. One novelty of this paper is to analyze how to design the option contract for a fresh agricultural product supply chain under two supply chain structures when the production cost and the loss rate are disrupted simultaneously. In addition to the effect of the cost and loss disruptions, this paper explores the effect of supply chain structure on the option coordination conditions, which is the most important innovation of this paper.

## 3. Supply chain coordination without disruptions

This paper considers a two-stage fresh agricultural product supply chain (*c*) consisted of one risk-neutral supplier (*s*) and one risk-neutral distributor (*d*). The supplier produces the fresh agricultural products at unit production cost *c*, and sells them to the distributor though option contracts. As is well known, option contracts have two parameters. One is the option price *o* paid by the distributor to the supplier for reserving one unit of the production capacity before the beginning of the selling season. The other is the exercise price *e* paid by the distributor to the supplier for exercising one unit of the purchased options during the selling season. The distributor faces stochastic market demand *D* and sells the fresh agricultural products to the customers at unit retail price *p*. Market demand *D* is a stochastic variable with cumulative distribution function (CDF) *F*(*x*) and probability density function (PDF) *f*(*x*). We define *q* to represent the quantity of products that the supplier produces, namely the quantity of options that the distributor purchases. We invite *β* (0<*β*<1) to represent the loss rate of products in the process of circulation. As a result, the quantity of options that the distributor exercises is *min*[*q*(1−*β*),*D*]. Without loss of generality, we assume that the unsold fresh agricultural products have no salvage value at the end of the selling season. We also assume *p*(1−*β*)>*o*+*e*(1−*β*)>*c* to ensure the profits for the supplier and the distributor.

Without disruptions, the expected profit of the supplier is

$$\Pi_{s}(q)=oq+eE\{min[q(1-\beta ),D]\}-cq$$ (1)

The expected profit of the distributor is

$$\Pi_{d}(q)=pE\{min[q(1-\beta ),D]\}-oq-eE\{min[q(1-\beta ),D]\}$$ (2)

Thus, the expected profit of the supply chain is

$$\Pi_{c}(q)=pE\{min[q(1-\beta ),D]\}-cq$$ (3)

In the following, we first investigate the optimal decision of the integrated supply chain without disruptions, and then discuss the coordination of the decentralized supply chain with the supplier-led supply chain structure (represented by subscript “1”) and the distributor-led supply chain structure (represented by subscript “2”) without disruptions.

### 3.1 Integrated decision without disruptions

Let q* be the optimal solution of Eq (3), i.e., q* = arg *max*_*q*≥0_ Π_*c*_(*q*). After calculating the derivations of Π_*c*_(*q*) with respective to *q*, it is verified that Π_*c*_(*q*) is a concave function of *q*. Without disruptions, the optimal decision of the integrated supply chain is given by

$$q^{*}=\frac{1}{1-\beta }F^{-1}\left[1-\frac{c}{p(1-\beta )}\right]$$ (4)

From the above, it is found that without disruptions the optimal decision of the integrated supply chain is decreasing in *c*. That is, a higher value of the production cost will induce a decreased supply quantity of the supply chain.

### 3.2 Supplier-led supply chain coordination without disruptions

We first consider that the supplier is the leader and the distributor is the follower. According to Liu *et al*. [37], the sequence of events in the supplier-led supply chain structure is given as follows. Before the beginning of the selling season, the supplier offers option contract to the distributor, and determines the supply policy. After that, the distributor places an option order by paying a unit option price per unit to the supplier based on the contract terms and the supplier’s supply decision. During the selling season, the distributor chooses the quantity of options to be exercised by paying a unit exercise price per unit to the supplier based on the realized demand.

Following Jeuland and Shugan [42], the coordination of the supplier-led supply chain can be achievable under option contract when the profit function of the distributor is the affine transformation of the profit function of the supply chain. Let *ϕ* (0<*ϕ*<1) denote the profit allocation coefficient. By comparing Eq (2) against Eq (3), the following result can be derived.

**Proposition 1** Without disruptions, the supplier-led supply chain can be coordinated by option contract when $\left\{ \begin{array}{l} o_{1}=\phi c\\ e_{1}=(1-\phi )p \end{array}\right.$.

This proposition suggests that without disruptions the coordination of the supplier-led supply chain is independent on the distribution of market demand. The parameters satisfying the condition in proposition 1 conform to the “*distribution-free*” criterion for evaluating the applicability of a coordinating contract. In addition, it is derived that *o*_1_ is increasing in *ϕ* while *e*_1_ is decreasing in *ϕ* under the coordinating contract. That is, as *ϕ* is increased, the value of *o*_1_ is set at a higher level while the value of *e*_1_ is set at a lower level.

### 3.3 Distributor-led supply chain coordination without disruptions

We now consider that the supplier is the follower and the distributor is the leader. According to Liu *et al*. [37], the sequence of events in the distributor-led supply chain structure is as follows. Before the beginning of the selling season, the distributor offers option contract to the supplier, and decides the option order policy. After that, the supplier determines the supply quantity based on the contract terms and the distributor’s ordering decision. The distributor commits to pay a unit option price per unit for the supplier’s output. During the selling season, the distributor chooses the quantity of options to be exercised by paying a unit exercise price per unit to the supplier based on the realized demand.

Similarly, following Jeuland and Shugan [42], the coordination of the distributor-led supply chain can be achievable under option contract when the profit function of the supplier is the affine transformation of the profit function of the supply chain. By comparing Eq (1) against Eq (3), the following result can be derived.

**Proposition 2** Without disruptions, the distributor-led supply chain can be coordinated by option contract when $\left\{ \begin{array}{l} o_{2}=(1-\phi )c\\ e_{2}=\phi p \end{array}\right.$.

This proposition indicates that without disruptions the coordination of the distributor-led supply chain is independent on the distribution of market demand. This result is in line with that of proposition 1. Thus, it is concluded that without disruptions the option parameters of the coordinating contracts in the supplier-led and distributor-led supply chains are unrelated to the distribution of market demand. In addition, it is derived that *o*_2_ is decreasing in *ϕ* while *e*_2_ is increasing in *ϕ* under the coordinating contract. That is, as *ϕ* is increased, the value of *o*_2_ is determined at a lower level while the value of *e*_2_ is determined at a higher level in the distributor-led supply chain. This result is contrary to that of Proposition 1.

## 4. Supply chain coordination with disruptions

In practice, the production cost and the loss rate are often disrupted by the emergencies. We assume that the production cost changes from *c* to *c*+Δ*c* and the loss rate changes from *β* to *β*+Δ*β*, where Δ*c*>0 (<0) represents an increased (decreased) production cost and Δ*β*>0 (<0) represents an increased (decreased) loss rate. The disruptions of production cost and loss rate may make the original optimal decision of the integrated supply chain q* become suboptimal [20,21,23], which may result in the deviation quantity associated with the deviation penalty. We define $\bar{q}$ to represent the quantity of products that the supplier produces under disruptions, namely the quantity of options that the distributor purchases under disruptions. We invite *c*_*u*_ (>0) to denote unit penalty cost for the increased quantity ${\left(\bar{q}-q^{*}\right)}^{+}$ and *c*_*s*_ (>0) to denote unit penalty cost for the decreased quantity ${\left(q^{*}-\bar{q}\right)}^{+}$.

With disruptions, the expected profit of the supplier is

$${\bar{\Pi }}_{s}(\bar{q})=o\bar{q}+eE\{min[\bar{q}(1-\beta -\Delta \beta ),D]\}-(c+\Delta c)\bar{q}-c_{u}{\left(\bar{q}-q^{*}\right)}^{+}-c_{s}{\left(q^{*}-\bar{q}\right)}^{+}$$ (5)

and the expected profit of the distributor is

$${\bar{\Pi }}_{d}(\bar{q})=pE\{min[\bar{q}(1-\beta -\Delta \beta ),D]\}-o\bar{q}-eE\{min[\bar{q}(1-\beta -\Delta \beta ),D]\}$$ (6)

Thus, the expected profit of the supply chain is

$${\bar{\Pi }}_{c}(\bar{q})=pE\{min[\bar{q}(1-\beta -\Delta \beta ),D]\}-(c+\Delta c)\bar{q}-c_{u}{\left(\bar{q}-q^{*}\right)}^{+}-c_{s}{\left(q^{*}-\bar{q}\right)}^{+}$$ (7)

In the following, we first investigate the optimal decision of the integrated supply chain with disruptions, and then discuss the coordination of the decentralized supply chain with two supply chain structures with disruptions.

### 4.1 Integrated decision with disruptions

Let ${\bar{q}}^{*}$ be the optimal solution of Eq (7), i.e., ${\bar{q}}^{*}=\arg\;{max}_{\bar{q}\geq 0}{\bar{\Pi }}_{c}(\bar{q})$. Then, the following result can be derived.

**Lemma 1** ${\bar{q}}^{*}\geq q^{*}$ when Δ*β*>0 and $\Delta c<\frac{c_{s}(1-\beta )-c\Delta \beta }{1-\beta };\;{\bar{q}}^{*}\leq q^{*}$ when Δ*β*<0 and $\Delta c>-\frac{c_{u}(1-\beta )+c\Delta \beta }{1-\beta }$.

Based on the above analysis, the following result can be derived.

**Theorem 1** Under disruptions, the optimal supply quantity of the supply chain is

$${\bar{q}}^{*}=\left\{ \begin{array}{l} {\bar{q}}^{\lambda }\;if\;{\bar{q}}^{\lambda }\geq q^{*}\\ q^{*}\;if\;{\bar{q}}^{\lambda }<q^{*}<{\bar{q}}^{\gamma }\\ {\bar{q}}^{\gamma }\;if\;{\bar{q}}^{\gamma }\leq q^{*} \end{array}\right.$$

where ${\bar{q}}^{\lambda }=\frac{1}{1-\beta -\Delta \beta }F^{-1}\left[1-\frac{c+\Delta c+c_{u}}{p(1-\beta -\Delta \beta )}\right]$ and ${\bar{q}}^{\gamma }=\frac{1}{1-\beta -\Delta \beta }F^{-1}\left[1-\frac{c+\Delta c-c_{s}}{p(1-\beta -\Delta \beta )}\right]$.

From the above, it is found that the disruptions of production cost and loss rate have the following impact on the integrated decision of the supply chain. For the case of Δ*β*>0 and $\Delta c<\frac{c_{s}(1-\beta )-c\Delta \beta }{1-\beta }$ and the case of Δ*β*<0 and $\Delta c>-\frac{c_{u}(1-\beta )+c\Delta \beta }{1-\beta }$, it is optimal for the supply chain to adjust the decision. In contrast, for the case of Δ*β*>0 and $\Delta c>\frac{c_{s}(1-\beta )-c\Delta \beta }{1-\beta }$ and the case of Δ*β*<0 and $\Delta c<-\frac{c_{u}(1-\beta )+c\Delta \beta }{1-\beta }$, it is optimal for the supply chain to keep the original decision.

### 4.2 Supplier-led supply chain coordination with disruptions

We first explore whether the original option contract without disruptions {*o*_1_, *e*_1_} can be used for coordinating the supplier-led supply chain under disruptions. The following result can be derived.

**Proposition 3** With disruptions, the coordination of the supplier-led supply chain will be broken off if the original option contract without disruptions {*o*_1_, *e*_1_} is applied.

This proposition indicates that with disruptions the profit function of the distributor is not the affine transformation of the profit function of the supply chain. Thus, option contract need be redesigned to achieve the coordination of the supplier-led supply chain under disruptions. Then, the following result can be derived.

**Proposition 4** With disruptions, the supplier-led supply chain can be coordinated by the modified option contract when $\left\{ \begin{array}{l} o_{1}^{\prime }=o_{1}+\phi [\Delta c+\frac{c_{u}{\left(\bar{q}-q^{*}\right)}^{+}+c_{s}{\left(q^{*}-\bar{q}\right)}^{+}}{\bar{q}}]\\ e_{1}^{\prime }=e_{1} \end{array}\right.$.

This proposition shows that with disruptions the coordination of the supplier-led supply chain is achieved with knowledge of the distribution of market demand. This result is contrary to that of Proposition 1. In addition, it is derived that the value of $e_{1}^{\prime }$ can be specified for a fixed *ϕ* with a pre-negotiated *p*, while the value of $o_{1}^{\prime }$ cannot be specified for a fixed *ϕ* with a pre-negotiated *c* and an additional $\Delta c+\frac{c_{u}{\left(\bar{q}-q^{*}\right)}^{+}+c_{s}{\left(q^{*}-\bar{q}\right)}^{+}}{\bar{q}}$. This result is different from that of proposition 1.

### 4.3 Distributor-led supply chain coordination with disruptions

Similarly, we first explore whether the original option contract without disruptions {*o*_2_, *e*_2_} can be used for coordinating the distributor-led supply chain under disruptions. Based on the above analysis, the following result can be derived.

**Proposition 5** With disruptions, the coordination of the distributor-led supply chain will be broken off if the original option contract without disruptions {*o*_2_, *e*_2_} is applied.

This proposition indicates that with disruptions the profit function of the supplier is not the affine transformation of the profit function of the supply chain. Therefore, option contract need be redesigned to achieve the coordination of the distributor-led supply chain under disruptions. Then, the following result can be derived.

**Proposition 6** With disruptions, the distributor-led supply chain can be coordinated by modified option contract when $\left\{ \begin{array}{l} o_{2}^{\prime }=o_{2}+(1-\phi )[\Delta c+\frac{c_{u}{\left(\bar{q}-q^{*}\right)}^{+}+c_{s}{\left(q^{*}-\bar{q}\right)}^{+}}{\bar{q}}]\\ e_{2}^{\prime }=e_{2} \end{array}\right.$.

This proposition indicates that with disruptions the coordination of the distributor-led supply chain is achieved with knowledge of the distribution of market demand. This result is contrary to that of Proposition 2. However, in combination with Proposition 4, it is concluded that with disruptions the option parameters of the coordinating contracts in the supplier-led and distributor-led supply chain structures are related to the distribution of market demand. In addition, the value of $e_{2}^{\prime }$ can be fixedly determined for a pre-negotiated *ϕ* with a specific *p*, while the value of $o_{2}^{\prime }$ can be arbitrarily determined for a pre-negotiated *ϕ* with a specific *c* and an additional $\Delta c+\frac{c_{u}{\left(\bar{q}-q^{*}\right)}^{+}+c_{s}{\left(q^{*}-\bar{q}\right)}^{+}}{\bar{q}}$. This result is different from that of Proposition 2. However, in combination with Proposition 4, it is concluded that in two coordinating contracts the exercise price is still at the original level without disruptions while the option price deviates from the initial level without disruptions under two supply chain structures.

## 5. Supplier-led structure vs. Distributor-led structure

This section studies the impact of supply chain structure on the option coordination conditions with disruptions. Then, the following result can be derived.

**Proposition 7** With disruptions, the relationships of the coordination conditions in two supply chain structures are related as follows.

1. If *ϕ*≥0.5, then $o_{1}^{\prime }\geq o_{2}^{\prime }$; if *ϕ*≤0.5, then $o_{1}^{\prime }\leq o_{2}^{\prime }$.
2. If *ϕ*≥0.5, then $e_{1}^{\prime }\leq e_{2}^{\prime }$; if *ϕ*≤0.5, then $e_{1}^{\prime }\geq e_{2}^{\prime }$.

This proposition indicates when the profit allocation coefficient exceeds the threshold (*ϕ* = 0.5), the option price is determined at a higher value in the supplier-led supply chain structure than in the distributor-led supply chain structure, while at the same time, the exercise price is determined at a lower value in the supplier-led supply chain structure than in the distributor-led supply chain structure. On the contrary, when the profit allocation coefficient falls behind the threshold (*ϕ* = 0.5), the option price is determined at a lower value in the supplier-led supply chain structure than in the distributor-led supply chain structure, while at the same time, the exercise price is determined at a higher value in the supplier-led supply chain structure than in the distributor-led supply chain structure.

## 6. Numerical example

This section gives a numerical example to illustrate the effect of the cost and loss disruptions on the integrated decision of the supply chain and the parameters of the coordinating contracts and highlight the difference between the coordinated supply chain and the uncoordinated supply chain. Partial parameter values are set as *p* = 45, *c* = 6, *β* = 0.2, *c*_*u*_ = 1, *c*_*s*_ = 2 and *x*~*N*(50,800). All the computational results are given in Table 1.

**Table 1:** The parameters, the decisions and the profits under disruptions.

Case	Δ*β*	Δ*c*	${\bar{q}}^{*}$	Supplier-led supply chain							Distributor-led supply chain						
				$\left\{o_{1}^{\prime },e_{1}^{\prime }\right)$	Original option contract			Modified option contract			$\left\{o_{2}^{\prime },e_{2}^{\prime }\right)$	Original option contract			Modified option contract		
					${\bar{\Pi }}_{s}^{1}$	${\bar{\Pi }}_{d}^{1}$	${\bar{\Pi }}_{c}^{1}$	${\bar{\Pi }}_{s}^{1}$	${\bar{\Pi }}_{d}^{1}$	${\bar{\Pi }}_{c}^{1}$		${\bar{\Pi }}_{s}^{2}$	${\bar{\Pi }}_{d}^{2}$	${\bar{\Pi }}_{c}^{2}$	${\bar{\Pi }}_{s}^{2}$	${\bar{\Pi }}_{d}^{2}$	${\bar{\Pi }}_{c}^{2}$
Ⅰ	0.06	-2.2	1216	{1.58,27}	5692	2263	7955	4829	3219	8048	{2.37,18}	4815	2948	7763	3219	4829	8048
	0.07	-2.0	1194	{1.65,27}	5433	2228	7661	4636	3091	7727	{2.48,18}	4494	3046	7540	3091	4636	7727
	0.08	-1.8	1172	{1.73,27}	5171	2193	7364	4444	2963	7407	{2.59,18}	4186	3107	7293	2963	4444	7407
II	0.06	2.2	1030	{3.28,27}	1070	2263	3333	2033	1356	3389	{4.92,18}	588	2524	3112	1356	2033	3389
	0.07	2.0	1030	{3.20,27}	1221	2228	3449	2104	1403	3507	{4.80,18}	659	2597	3256	1403	2104	3507
	0.08	1.8	1030	{3.12,27}	1373	2193	3566	2174	1450	3624	{4.68,18}	738	2663	3401	1450	2174	3624
III	-0.06	-2.2	1030	{1.52,27}	6157	2635	8792	5309	3539	8848	{2.28,18}	5715	1539	7254	3539	5309	8848
	-0.07	-2.0	1030	{1.60,27}	5989	2662	8651	5226	3484	8710	{2.40,18}	5326	2435	7761	3484	5226	8710
	-0.08	-1.8	1030	{1.68,27}	5822	2690	8512	5143	3428	8571	{2.52,18}	4985	2965	7950	3428	5143	8571
IV	-0.06	2.2	982	{3.32,27}	1691	2635	4326	2597	1731	4328	{4.98,18}	379	3945	4324	1731	2597	4328
	-0.07	2.0	998	{3.23,27}	1933	2662	4595	2758	1838	4596	{4.84,18}	602	3993	4595	1838	2758	4596
	-0.08	1.8	1013	{3.13,27}	2173	2690	4863	2919	1946	4865	{4.70,18}	833	4027	4860	1946	2919	4865

In Table 1, for the first case, when both the loss rate and the production cost increase and satisfies Δ*β*>0 and $\Delta c<\frac{c_{s}(1-\beta )-c\Delta \beta }{1-\beta }$, the optimal supply quantity of the supply chain is raised to a higher level and the option prices in two coordinating contracts are determined at a lower value. For the second case, when the loss rate increases and the production cost decreases and satisfies Δ*β*>0 and $\Delta c>\frac{c_{s}(1-\beta )-c\Delta \beta }{1-\beta }$, the optimal supply quantity of the supply chain is maintained at the original level and the option prices in two coordinating contracts are set at a higher value. For the third case, when the loss rate decreases and the production cost increases and satisfies Δ*β*<0 and $\Delta c<-\frac{c_{u}(1-\beta )+c\Delta \beta }{1-\beta }$, the optimal supply quantity of the supply chain is maintained at the original level and the option prices in two coordinating contracts are set at a lower value. For the fourth case, when both the loss rate and the production cost decrease and satisfies Δ*β*<0 and $\Delta c>-\frac{c_{u}(1-\beta )+c\Delta \beta }{1-\beta }$, the optimal supply quantity of the supply chain is fallen to a lower level and the option prices in two coordinating contracts are determined at a higher value.

Table 1 suggests that in two coordinating contracts the values of the option prices need be adjusted while the values of the exercise prices do not need be adjusted under disruptions. When the production cost and the loss rate are disrupted simultaneously, it is unfavorable to use the original coordinating contracts without disruptions to coordinate the disrupted supply chain under two supply chain structures. In addition, the disrupted supply chain will obtain more profit in the modified coordinating contracts than in the original coordinating contracts under disruptions. Therefore, it is necessary to employ the modified coordinating contracts to coordinate the disrupted supply chain under two supply chain structures.

## 7. Conclusions

This paper derives the option coordination conditions for a fresh agricultural product supply chain with a supplier and a distributor under two supply chain structures when the production cost and the loss rate are disrupted simultaneously. In addition to the impact of the cost and loss disruptions, this paper explores the impact of supply chain structure on the coordination conditions. The results show that when the degree of the disruptions is within a certain range, the optimal decision of the supply chain does not need to be changed while only the option price should be changed for the coordination under two supply chain structures. When the degree of the disruptions is beyond a certain range, both the optimal decision of the supply chain and the option price need be changed for the coordination at the same time under two supply chain structures. Moreover, the coordination of the disrupted supply chain is achieved with knowledge of the distribution of market demand. In two coordinating contracts for the disrupted supply chain, the exercise price still stays at the original level without disruptions while the option price deviates from the original level without disruptions. Furthermore, the relationships of the option coordination conditions in two supply chain structures depend on the value of the profit allocation coefficient. When the profit allocation coefficient exceeds (falls behind) the threshold, the option price is set at a higher (lower) value in the supplier-led supply chain structure than in the distributor-led supply chain structure, while the exercise price is set at a lower (higher) value in the supplier-led supply chain structure than in the distributor-led supply chain structure. Finally, the disrupted supply chain with any supply chain structure will obtain more profit in the modified coordinating contracts than in the original coordinating contracts without disruptions.

Managerial implications are provided as follows. First, we derive the option coordination conditions for the fresh agricultural product supply chain without and with disruptions, which is helpful for the members to improve their performances under various situations. Moreover, we explore the impact of the cost and loss disruptions on the option coordination conditions for the fresh agricultural product supply chain, which is helpful for the members to design the coordinating contract rationally to obtain more profit. Finally, we investigate the impact of supply chain structure on the option coordination conditions for the fresh agricultural product supply chain, which enables the members to make strategic decisions toward sustainable management.

Several extensions are worthy of being considered in the future study. In practice, a fresh agricultural product supply chain is consisted of multiple suppliers and multiple distributors. It would be interesting to analyze the coordination of a fresh agricultural product supply chain with a more complicated membership. Moreover, the information is assumed to be symmetric between the supplier and the distributor. It would be interesting to investigate the coordination of a fresh agricultural product supply chain under asymmetric information.

## Appendix

**Proof of Proposition 1** By substituting {*o*_1_, *e*_1_} into Eq (2), we get $\Pi_{d}(q)=\phi [p(1-\beta )-c]q-\phi p\int_{0}^{q(1-\beta )}F(x)dx=\phi \Pi_{c}(q)$, which is the affine function of the expected profit of the supply chain. This completes the proof.

**Proof of Proposition 2** By substituting {*o*_2_, *e*_2_} into Eq (1), we get Π_*s*_(*q*) = [*e*_2_(1−*β*)+*o*_2_−*c*]*q*−*e*_2_(*q*), which is the affine function of the expected profit of the supply chain. This completes the proof.

**Proof of Lemma 1** Suppose that ${\bar{q}}^{*}<q^{*}$ if Δ*β*>0 and $\Delta c<\frac{c_{s}(1-\beta )-c\Delta \beta }{1-\beta }$. Then, ${\bar{q}}^{*}=\arg\;{max}_{\bar{q}\geq 0}{\bar{\Pi }}_{c}(\bar{q})$, where ${\bar{\Pi }}_{c}(\bar{q})=[p(1-\beta -\Delta \beta )-c-\Delta c]\bar{q}-p\int_{0}^{\bar{q}(1-\beta -\Delta \beta )}F(x)dx-c_{s}(q^{*}-\bar{q})$. By calculating the derivations of ${\bar{\Pi }}_{c}(\bar{q})$ with respective to $\bar{q}$, we get $\frac{d{\bar{\Pi }}_{c}(\bar{q})}{d\bar{q}}=[p(1-\beta -\Delta \beta )-c-\Delta c+c_{s}]-p(1-\beta -\Delta \beta )F[\bar{q}(1-\beta -\Delta \beta )],\;\frac{d^{2}{\bar{\Pi }}_{c}(\bar{q})}{d{\bar{q}}^{2}}=-p{\left(1-\beta -\Delta \beta \right)}^{2}f[\bar{q}(1-\beta -\Delta \beta )]<0$. It means that ${\bar{\Pi }}_{c}(\bar{q})$ is a concave function of $\bar{q}$. Let $\frac{d{\bar{\Pi }}_{c}(\bar{q})}{d\bar{q}}=0$, we get $F[{\bar{q}}^{*}(1-\beta -\Delta \beta )]=1-\frac{c+\Delta c-c_{s}}{p(1-\beta -\Delta \beta )}$. Since $\Delta c<\frac{c_{s}(1-\beta )-c\Delta \beta }{1-\beta }$, we get $F[{\bar{q}}^{*}(1-\beta -\Delta \beta )]>F[q^{*}(1-\beta )]$. Since Δ*β*>0, we get $F[{\bar{q}}^{*}(1-\beta )]>F[{\bar{q}}^{*}(1-\beta -\Delta \beta )]$. So, it follows that $F[{\bar{q}}^{*}(1-\beta )]>F[q^{*}(1-\beta )]$, i.e., ${\bar{q}}^{*}>q^{*}$, which is a contradiction to the assumption ${\bar{q}}^{*}<q^{*}$. Thus, we have ${\bar{q}}^{*}\geq q^{*}$ if Δ*β*>0 and $\Delta c<\frac{c_{s}(1-\beta )-c\Delta \beta }{1-\beta }$. Similarly, we have ${\bar{q}}^{*}\leq q^{*}$ if Δ*β<*0 and $\Delta c>-\frac{c_{u}(1-\beta )+c\Delta \beta }{1-\beta }$. This completes the proof.

**Proof of Theorem 1** If $\bar{q}>q^{*}$, the expected profit of the supply chain is ${\bar{\Pi }}_{c}^{\lambda }(\bar{q})=[p(1-\beta -\Delta \beta )-c-\Delta c]\bar{q}-c_{u}(\bar{q}-q^{*})-p\int_{0}^{\bar{q}(1-\beta -\Delta \beta )}F(x)dx$. By calculating the derivations of ${\bar{\Pi }}_{c}^{\lambda }(\bar{q})$ with respective to $\bar{q}$, we get $\frac{d{\bar{\Pi }}_{c}^{\lambda }(\bar{q})}{d\bar{q}}=[p(1-\beta -\Delta \beta )-c-\Delta c-c_{u}]-p(1-\beta -\Delta \beta )F[\bar{q}(1-\beta -\Delta \beta )],\;\frac{d^{2}{\bar{\Pi }}_{c}^{\lambda }(\bar{q})}{d{\bar{q}}^{2}}=-p{\left(1-\beta -\Delta \beta \right)}^{2}f[\bar{q}(1-\beta -\Delta \beta )]<0$. It indicates that ${\bar{\Pi }}_{c}^{\lambda }(\bar{q})$ is a concave function of $\bar{q}$. Let ${\bar{q}}^{\lambda }$ satisfy the corresponding first order optimality condition, i.e., ${\bar{q}}^{\lambda }=\frac{1}{1-\beta -\Delta \beta }F^{-1}\left[1-\frac{c+\Delta c+c_{u}}{p(1-\beta -\Delta \beta )}\right]$. Based on the monotonic property of ${\bar{\Pi }}_{c}^{\gamma }(\bar{q})$, if ${\bar{q}}^{\lambda }>q^{*},\;{\bar{q}}^{\lambda }$ is the local optimum point of ${\bar{\Pi }}_{c}^{\lambda }(\bar{q})$, i.e., ${\bar{q}}^{\lambda }=\arg\;{max}_{\bar{q}>q^{*}}{\bar{\Pi }}_{c}^{\lambda }(\bar{q})$. If ${\bar{q}}^{\lambda }<q^{*}$, ${\bar{\Pi }}_{c}^{\lambda }(\bar{q})$ is strictly decreasing in the range [q*,+∞) and q* is the local optimum point of ${\bar{\Pi }}_{c}^{\lambda }(\bar{q})$, i.e., $q^{*}=\arg\;{max}_{\bar{q}>q^{*}}{\bar{\Pi }}_{c}^{\lambda }(\bar{q})$.

If $\bar{q}<q^{*}$, the expected profit of the supply chain is ${\bar{\Pi }}_{c}^{\gamma }(\bar{q})=[p(1-\beta -\Delta \beta )-c-\Delta c]\bar{q}-c_{s}{\left(q^{*}-\bar{q}\right)}^{+}-p\int_{0}^{\bar{q}(1-\beta -\Delta \beta )}F(x)dx$. By calculating the derivations of ${\bar{\Pi }}_{c}^{\gamma }(\bar{q})$ with respective to $\bar{q}$, we get $\frac{d{\bar{\Pi }}_{c}^{\gamma }(\bar{q})}{d\bar{q}}=[p(1-\beta -\Delta \beta )-c-\Delta c+c_{s}]-p(1-\beta -\Delta \beta )F[\bar{q}(1-\beta -\Delta \beta )],\;\frac{d^{2}{\bar{\Pi }}_{c}^{\gamma }(\bar{q})}{d{\bar{q}}^{2}}=-p{\left(1-\beta -\Delta \beta \right)}^{2}f[\bar{q}(1-\beta -\Delta \beta )]<0$. It indicates that ${\bar{\Pi }}_{c}^{\gamma }(\bar{q})$ is a concave function of $\bar{q}$. Let ${\bar{q}}^{\gamma }$ satisfy the corresponding first order optimality condition, i.e., ${\bar{q}}^{\gamma }=\frac{1}{1-\beta -\Delta \beta }F^{-1}\left[1-\frac{c+\Delta c-c_{s}}{p(1-\beta -\Delta \beta )}\right]$. Based on the monotonic property of ${\bar{\Pi }}_{c}^{\gamma }(\bar{q})$, if ${\bar{q}}^{\gamma }<q^{*},\;{\bar{q}}^{\gamma }$ is the local optimum point of ${\bar{\Pi }}_{c}^{\gamma }(\bar{q})$, i.e., ${\bar{q}}^{\gamma }=\arg\;{max}_{\bar{q}<q^{*}}{\bar{\Pi }}_{c}^{\gamma }(\bar{q})$. If ${\bar{q}}^{\gamma }>q^{*},\;{\bar{\Pi }}_{c}^{\gamma }(\bar{q})$ is strictly increasing in the range (0,q*] and q* is the local optimum point of ${\bar{\Pi }}_{c}^{\gamma }(\bar{q})$, i.e., $q^{*}=\arg\;{max}_{\bar{q}<q^{*}}{\bar{\Pi }}_{c}^{\gamma }(\bar{q})$.

If ${\bar{q}}^{\lambda }\geq q^{*},\;{\bar{q}}^{\lambda }$ is the local optimum point of ${\bar{\Pi }}_{c}(\bar{q})$ in the range [q*,+∞) while q* is the local optimum point of ${\bar{\Pi }}_{c}(\bar{q})$ in (0,q*]. Therefore, the maximum value of ${\bar{\Pi }}_{c}(\bar{q})$ is ${\bar{\Pi }}_{c}({\bar{q}}^{\lambda })$ or ${\bar{\Pi }}_{c}({\bar{q}}^{*})$. Obviously, we have ${\bar{\Pi }}_{c}({\bar{q}}^{\lambda })\geq {\bar{\Pi }}_{c}({\bar{q}}^{*})$. Therefore, if ${\bar{q}}^{\lambda }\geq q^{*},\;{\bar{q}}^{\lambda }$ is the global optimum point of ${\bar{\Pi }}_{c}(\bar{q})$. Similarly, if ${\bar{q}}^{\gamma }\leq q^{*},\;{\bar{q}}^{\gamma }$ is the global optimum point of ${\bar{\Pi }}_{I}(\bar{q})$.

In conclusion, if ${\bar{q}}^{\lambda }\geq q^{*}$, then ${\bar{q}}^{*}={\bar{q}}^{\lambda }$; if ${\bar{q}}^{\lambda }<q^{*}<{\bar{q}}^{\gamma }$, then ${\bar{q}}^{*}=q^{*}$; if ${\bar{q}}^{\gamma }\leq q^{*}$, then ${\bar{q}}^{*}={\bar{q}}^{\gamma }$. This completes the proof.

**Proof of Proposition 3** By substituting {*o*_1_, *e*_1_} into Eq (6), we get ${\bar{\Pi }}_{d}(\bar{q})=\phi [p(1-\beta -\Delta \beta )-c]\bar{q}-\phi p\int_{0}^{\bar{q}(1-\beta -\Delta \beta )}f(x)dx=\phi {\bar{\Pi }}_{c}(\bar{q})+\phi [\Delta c\bar{q}+c_{u}{\left(\bar{q}-q^{*}\right)}^{+}+c_{s}{\left(q^{*}-\bar{q}\right)}^{+}]$, which is not the affine function of the expected profit of the supply chain. This completes the proof.

**Proof of Proposition 4** By substituting $\left\{o_{1}^{\prime },e_{1}^{\prime }\right\}$ into Eq (6), we get ${\bar{\Pi }}_{d}(\bar{q})=\phi \left[p(1-\beta -\Delta \beta )-c-\Delta c-\frac{c_{u}{\left(\bar{q}-q^{*}\right)}^{+}+c_{s}{\left(q^{*}-\bar{q}\right)}^{+}}{\bar{q}}\right]\bar{q}-\phi p\int_{0}^{\tilde{q}(1-\beta -\Delta \beta )}F(x)dx=\phi {\bar{\Pi }}_{c}(\bar{q})$, which is the affine of the expected profit of the supply chain. This completes the proof.

**Proof of Proposition 5** By substituting {*o*_2_, *e*_2_} into Eq (5), we get ${\bar{\Pi }}_{s}(\bar{q})=\left[\phi p(1-\beta -\Delta \beta )-\phi c-\Delta c-\frac{c_{u}{\left(\bar{q}-q^{*}\right)}^{+}+c_{s}{\left(q^{*}-\bar{q}\right)}^{+}}{\bar{q}}\right]\bar{q}-\phi p\int_{0}^{\bar{q}(1-\beta -\Delta \beta )}F(x)dx=\phi {\bar{\Pi }}_{c}(\bar{q})-(1-\phi )[\Delta c\bar{q}+c_{u}{\left(\bar{q}-q^{*}\right)}^{+}+c_{s}{\left(q^{*}-\bar{q}\right)}^{+}]$, which is not the affine function of the expected profit of the supply chain. This completes the proof.

**Proof of Proposition 6** By substituting $\left\{o_{2}^{\prime },e_{2}^{\prime }\right\}$ into Eq (5), we get ${\bar{\Pi }}_{s}(\bar{q})=\phi \left[p(1-\beta -\Delta \beta )-c-\Delta c-\frac{c_{u}{\left(\bar{q}-q^{*}\right)}^{+}+c_{s}{\left(q^{*}-\bar{q}\right)}^{+}}{\bar{q}}\right]\bar{q}-\phi p\int_{0}^{\bar{q}(1-\beta -\Delta \beta )}f(x)dx=\phi {\bar{\Pi }}_{c}(\bar{q})$, which is the affine function of the expected profit of the supply chain. This completes the proof.

**Proof of Proposition 7** From Propositions 4 and 6, we have $o_{1}^{\prime }-o_{2}^{\prime }=(2\phi -1)\left[c+\Delta c+\frac{c_{u}{\left(\bar{q}-q^{*}\right)}^{+}+c_{s}{\left(q^{*}-\bar{q}\right)}^{+}}{\bar{q}}\right]$. Therefore, it is easy to derive that $o_{1}^{\prime }\geq o_{2}^{\prime }$ if *ϕ*≥0.5 and $o_{1}^{\prime }\leq o_{2}^{\prime }$ if *ϕ*≤0.5. From Propositions 4 and 6, we have $e_{1}^{\prime }-e_{2}^{\prime }=(1-2\phi )p$. Therefore, it is easy to derive that $e_{1}^{\prime }\leq e_{2}^{\prime }$ if *ϕ*≥0.5 and $e_{1}^{\prime }\geq e_{2}^{\prime }$ if *ϕ*≤0.5. This completes the proof.
